# Genetic support for a quantitative trait nucleotide in the *ABCG2 *gene affecting milk composition of dairy cattle

**DOI:** 10.1186/1471-2156-8-32

**Published:** 2007-06-21

**Authors:** Hanne Gro Olsen, Heidi Nilsen, Ben Hayes, Paul R Berg, Morten Svendsen, Sigbjørn Lien, Theo Meuwissen

**Affiliations:** 1 BoviBank Ltd, Box 58, N-1431 Aas, Norway; 2 Department of Animal and Aquacultural Sciences, Norwegian University of Life Sciences, Box 5003, N-1432 Aas, Norway; 3 Animal Genetics and Genomics, Primary Industries Research Victoria, 475 Mickleham Rd, Attwood, Victoria, Australia 3049; 4 Centre for Integrative Genetics, Norwegian University of Life Sciences, Box 5003, N-1432 Aas, Norway; 5 Geno Breeding and AI organisation, Norwegian University of Life Sciences, Box 5003, N-1432 Aas, Norway

## Abstract

**Background:**

Our group has previously identified a quantitative trait locus (QTL) affecting fat and protein percentages on bovine chromosome 6, and refined the QTL position to a 420-kb interval containing six genes. Studies performed in other cattle populations have proposed polymorphisms in two different genes (*ABCG2 *and *OPN*) as the underlying functional QTL nucleotide. Due to these conflicting results, we have included these QTNs, together with a large collection of new SNPs produced from PCR sequencing, in a dense marker map spanning the QTL region, and reanalyzed the data using a combined linkage and linkage disequilibrium approach.

**Results:**

Our results clearly exclude the *OPN *SNP (*OPN_3907*) as causal site for the QTL. Among 91 SNPs included in the study, the *ABCG2 *SNP (*ABCG2_49*) is clearly the best QTN candidate. The analyses revealed the presence of only one QTL for the percentage traits in the tested region. This QTL was completely removed by correcting the analysis for *ABCG2_49*. Concordance between the sires' marker genotypes and segregation status for the QTL was found for *ABCG2_49 *only. The C allele of *ABCG2_49 *is found in a marker haplotype that has an extremely negative effect on fat and protein percentages and positive effect on milk yield. Of the 91 SNPs, *ABCG2_49 *was the only marker in perfect linkage disequilibrium with the QTL.

**Conclusion:**

Based on our results, OPN_3907 can be excluded as the polymorphism underlying the QTL. The results of this and other papers strongly suggest the [A/C] mutation in *ABCG2_49 *as the causal mutation, although the possibility that *ABCG2_49 *is only a marker in perfect LD with the true mutation can not be completely ruled out.

## Background

Since the first complete genome scan for quantitative trait loci (QTL) in cattle was performed in 1995 [1], a large number of genome scans have been performed, and QTL affecting important production, health and quality traits have been detected. In dairy cattle, most emphasis has been on detecting QTL for milk production traits, and QTL affecting one or more of the five milk production traits (i.e., milk yield, fat yield and percentage, and protein yield and percentage) have been detected on all autosomal chromosomes (for a review, see [2]). Bovine chromosome 6 (BTA6) seems to be among the bovine chromosomes that harbour the largest number of milk production QTL.

The major aim of QTL mapping is to characterize the gene(s) affecting the traits and identify the mutations underlying the genetic variation. This will yield important insight into the structure and function of the genome, and may also yield a valuable supplement to traditional animal breeding. However, the QTL position estimates obtained by most genome scans are usually too imprecise to identify the causal genes, with confidence intervals often ranging 20–30 cM and containing hundreds of genes. In order to narrow down these intervals, fine-mapping methods utilizing combined information from linkage and linkage disequilibrium have been developed (e.g. [3]). Such a method enabled us to map a QTL causing a major reduction in fat and protein percentages as well as a minor increase in milk yield to a 420 kb region on BTA6 [4]. By the use of comparative mapping we found that this area contained six genes; i.e. *ABCG2*, *PKD2*, *SPP1 *(also denoted *OPN*), *MEPE*, *IBSP *and *LAP3*. Two more recent studies have proposed polymorphisms in *ABCG2 *[5] and *OPN *[6] as the underlying functional sites based on mapping results in the Holstein breed, but the papers did not provide functional data to verify their findings. Comparing the arguments for each gene suggests that both mutations are equally probable [7]. In the present paper we have constructed a dense marker map spanning the QTL region in a different breed (Norwegian Red cattle), reanalyzed the traits using the combined linkage and linkage disequilibrium approach, and assessed the evidence for the *ABCG2*, *OPN *and other polymorphisms in our data.

## Results

**Figure 1:**
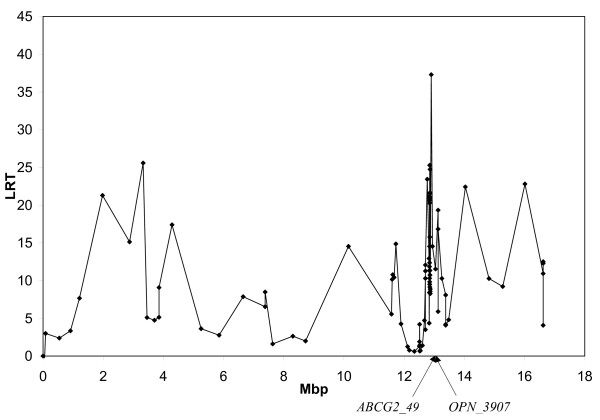
**Single QTL analysis for protein and fat percentages**. The Likelihood Ratio Test-statistic (LRT) is plotted on the y-axis and distances in megabases on the x-axis. Points illustrate bracket midpoints. Positions of *ABCG2_49 *and *OPN_3907 *are indicated.

**Figure 2:**
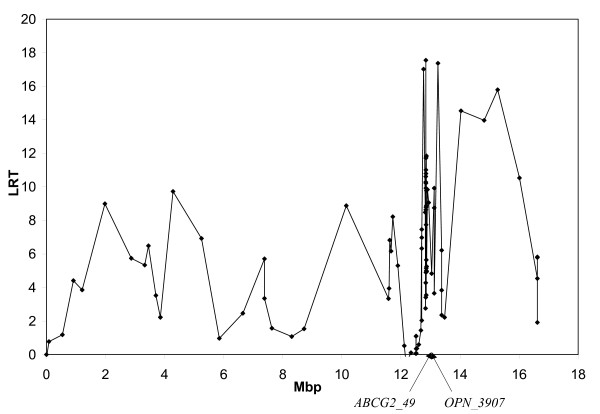
**Single QTL analysis for fat percentage**. The Likelihood Ratio Test-statistic (LRT) is plotted on the y-axis and distances in megabases on the x-axis. Points illustrate bracket midpoints. Positions of *ABCG2_49 *and *OPN_3907 *are indicated.

Initially, protein percentage and fat percentage were analyzed separately using a single-QTL approach. Results for the two traits are shown in Figure 1 and Figure 2, respectively. Both analyses revealed a large number of peaks across most of the genotyped area. For protein percentage, the highest likelihood ratio test statistic (LRT) was found for the interval between markers *PKD2_597 *and *OPN_3907 *(LRT = 37.3). High LRTs were also found for brackets *BMPR1B_1370 *– *AAFC02110660_20186 *(LRT = 25.6), *PKD2_610 *– *PKD2_349 *(LRT = 25.6), *PKD2_3610 *– *PKD2_3909 *(LRT = 24.7), and *BZ916464_460 *– *ABCG2_49 *(LRT = 23.4). For fat percentage, the highest peak was found for bracket *PKD2_1451 *– *PKD2_1349 *(LRT = 17.6), followed by *LAP3_581 *– *HCAPG_1119 *(LRT = 17.4) and *BZ916464_460 *– *ABCG2_49 *(LRT = 17.0). These results are in very good agreement with the findings of our previous study [4] that restricted the QTL position to an interval surrounded by *ABCG2 *and *LAP3*, but the QTL region was not further refined.

**Figure 3:**
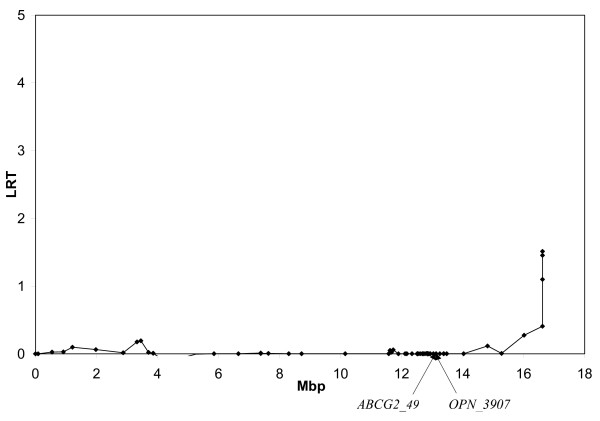
**Multi QTL analysis with *ABCG2_49 *included**. Analysis of protein percentage with *ABCG2_49 *included in the QTL model as a random effect. Positions of *ABCG2_49 *and *OPN_3907 *are indicated.

**Figure 4:**
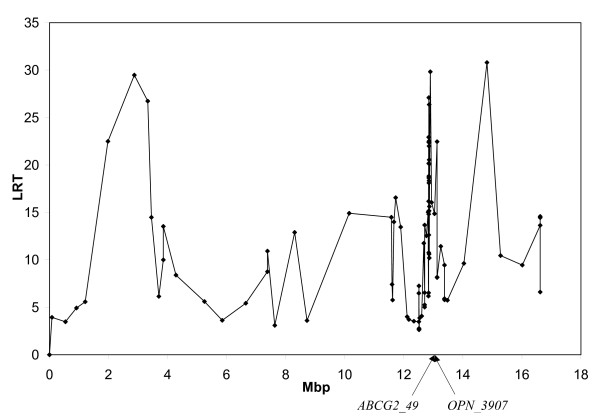
**Multi QTL analysis with *OPN_3907 *included**. Analysis of protein percentage with *OPN_3907 *included in the QTL model as a random effect. Positions of *ABCG2_49 *and *OPN_3907 *are indicated.

In order to refine the QTL position and investigate whether the large number of peaks was due to the presence of several QTL, a multi-QTL analysis was performed. We utilized the same method as for the single-QTL analyses, but included also a random effect of a specific marker. Markers situated in the region between *ABCG2 *and *LAP3 *and with high LRT from the single QTL analyses were included in the model one at a time, and the QTL analysis was repeated. Figure 3 shows the result of analyzing protein percentage with *ABCG2_49 *included as a random effect. This curve is almost completely flat, with no other peaks remaining at *OPN_3907 *or any other marker, indicating that most of the variation due to the QTL is explained by this marker. Figure 4 shows the effect of including *OPN_3907*. This curve is similar to the one from the single QTL analyses, indicating that this marker did not explain the QTL effect. Similar results were found for all other tested markers (not shown). These results suggest that the genetic variation is best explained by *ABCG2_49*, and that the other SNPs included in this study can be excluded as the causal QTL site.

The 'best LD marker test' revealed that *ABCG2_49 *and *OPN_3907 *showed the highest and second highest LD with the QTL (LRT = 62.4 and 20.9, respectively, for protein %).

Only two sires, which were paternal halfsibs, were found to be segregating for the QTL. These were also the only sires that carried the C-allele for *ABCG2_49*. The probability of concordance by chance, i.e. the probability that the two sires segregating for the QTL should also be heterozygous for the marker and all sires homozygous for the QTL should also be homozygous for the marker just by chance, is 0.0018. The *OPN_3907 *deletion was found in these two families as well as in two families that did not segregate for the QTL. None of the other markers showed any concordance with the QTL segregation status of the sires.

**Figure 5:**
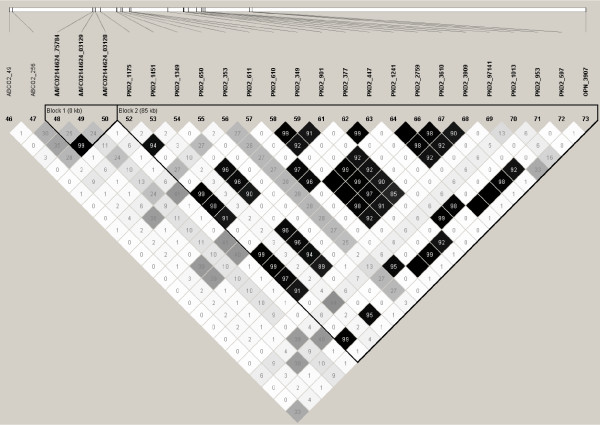
**Haplotype blocks and LD**. Haplotype block structure and LD (expressed as r^2^*100) between markers in the *ABCG2_49 *to *OPN_3907 *region.

**Figure 6:**
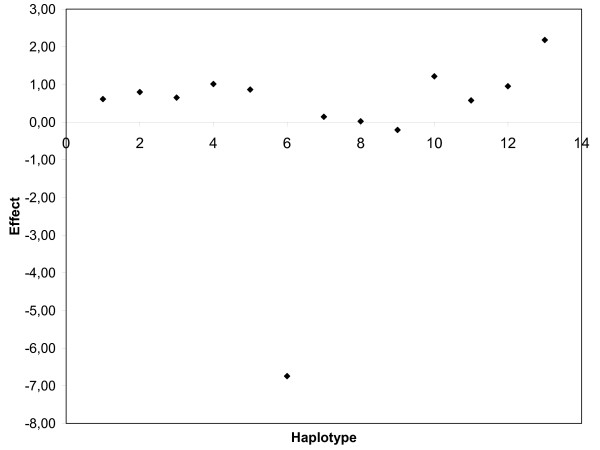
**Haplotype effects for protein percentage**. Haplotype ID numbers are plotted along the X-axis and the effects for protein percentage as measured in deviations from mean PTA on the Y-axis.

Next, we aimed to elucidate the haplotype block structure in the QTL region and identify haplotypes with major effect on the milk production traits. In general, extent of LD in the region was found to be much less than reported previously in cattle (eg. [8] and [9]). Average r^2 ^between markers situated 50 kb apart was approximately 0.2. This result limits our ability to clearly determine haplotype block structures and the number of haplotypes within each block to regions with a high SNP density. In our map, high SNP density can be found for the region between *ABCG2 *and *OPN *where we also have exact physical position of SNPs in bovine [GenBank:AJ871176]. Phases of animals were determined and imported into the Haploview program [10] for calculation of LD (r^2^) between markers and construction of haplotype blocks using the algorithm of Gabriel *et al*. [11]. As shown in Figure 5, one block of 85 kb, including all SNPs in *PKD2 *and *OPN_3907*, and one block including 3 SNPs in contig *AAFC02144624*, were formed. Notably, neither of the SNPs in *ABCG2 *was included into a block structure and the only SNP being in some degree of LD with *ABCG2_49 *was *OPN_3907 *(r^2 ^= 0.34). When forcing all SNPs into a single block (101 kb) we obtained 13 haplotypes with frequencies above 1% (Table 1). Notably, allele C of *ABCG2_49 *is present in only one of the haplotypes (haplotype 6) whereas the deletion in *OPN_3907 *is present in both haplotypes 4 and 6. Upon closer examination of haplotype 6, we found that it spanned a region of 6 Mb from *BZ912321_387 *to *HCAPG _339*, i.e., including approx. 60 markers. Haplotype 6 has an extremely negative effect on protein percentage (Figure 6) and fat percentage (not shown). The allele substitution effects correspond to 0.15% protein and 0.14% fat, which was very close to the effects found by Cohen-Zinder et al. [5]. Haplotype 6 also has an extremely negative effect on fat percentage, as well as an extremely positive effect on milk yield, but without any particular influence on fat and protein yields (not shown). Conversely haplotype 4, that also includes the deletion in *OPN_3907*, has no substantial effect on protein or fat percentages, which underpins our finding that *OPN_3907 *is not the causative polymorphism.

**Table 1:** Haplotypes and haplotype frequencies for markers between *ABCG2 *and *OPN*.

	Haplotype ID												
	
Marker name	1	2	3	4	5	6	7	8	9	10	11	12	13
ABCG2_49	A	A	A	A	A	C	A	A	A	A	A	A	A
ABCG2_256	G	G	G	G	G	G	A	A	A	G	A	A	G
AAFC02144624_75784	A	A	A	A	A	A	A	G	A	G	G	G	A
AAFC02144624_03129	G	G	A	G	A	G	A	A	A	A	A	A	G
AAFC02144624_03128	G	G	G	G	G	G	G	A	G	A	A	A	G
PKD2_746	G	G	G	G	G	G	G	A	G	A	A	A	G
PKD2_1175	A	A	A	A	D	A	A	A	A	A	A	A	A
PKD2_1451	G	A	A	A	A	A	A	A	A	A	A	A	A
PKD2_1349	T	T	T	T	A	T	T	T	T	T	T	T	T
PKD2_650	C	C	T	C	C	C	C	C	C	C	C	C	C
PKD2_353	C	C	C	C	T	C	C	C	T	T	T	C	T
PKD2_611	G	G	G	G	G	G	G	G	G	A	A	G	G
PKD2_610	T	T	T	T	A	T	T	T	T	T	T	T	T
PKD2_349	G	G	G	G	T	G	G	G	G	G	G	G	G
PKD2_383	G	G	G	G	G	G	G	G	G	G	G	G	A
PKD2_901	A	A	A	A	G	A	A	A	A	A	A	A	A
PKD2_377	T	T	T	T	T	T	T	T	T	A	A	T	T
PKD2_447	A	A	A	A	A	A	A	A	A	G	G	A	A
PKD2_1241	C	C	C	C	T	C	C	C	C	C	C	C	C
PKD2_2256	T	T	T	T	T	T	T	T	T	T	T	T	T
PKD2_2759	T	T	T	T	C	T	T	T	T	T	T	T	T
PKD2_3610	T	T	T	T	C	T	T	T	T	T	T	T	T
PKD2_3909	A	A	A	A	T	A	A	A	A	A	A	A	A
PKD2_97141	G	G	G	A	G	G	G	G	G	G	G	A	G
PKD2_1013	A	G	G	A	G	A	G	G	G	G	G	A	A
PKD2_953	C	C	C	C	C	C	C	C	C	T	T	C	C
PKD2_597	C	C	C	C	T	C	C	C	C	C	C	C	C
OPN_3907	T	T	T	D	T	D	T	T	T	T	T	T	T

Haplotype frequency	0.26	0.17	0.09	0.07	0.07	0.05	0.05	0.05	0.05	0.03	0.03	0.03	0.02

## Discussion

Former studies in Norwegian Red cattle have revealed a QTL affecting milk production between the markers *ABCG2 *and *LAP3 *on BTA6. The QTL showed a marked negative effect on protein- and fat percentages, as well as a minor positive effect on kg milk. The QTL was positioned to a 420-kb interval containing 6 genes; *ABCG2*, *PKD2*, *OPN *(*SPP1*), *MEPE*, *IBSP *and *LAP3 *[4]. At least three of the genes may be suggested as candidate genes for the QTL (i.e., *ABCG2*, *OPN *and *PKD2*). Schnabel et al. [6] suggested osteopontin (*OPN*, *SPP1*) as a strong candidate. This gene is shown to be necessary for normal mammary gland development and lactation in rodents. Schnabel and co-workers identified a deletion upstream of the *OPN *promoter (*OPN_3907*), in a region known to harbour tissue-specific osteopontin regulatory elements, which was in complete concordance with the segregation status of bulls segregating for the QTL. Cohen-Zinder et al. [5] suggested *ABCG2 *as the gene behind the milk QTL. *ABCG2 *is responsible for the active secretion of clinically and toxicologically important substrates into the milk of cows, mice and humans [12], and is thus an interesting functional candidate. Several SNPs have been identified in the *ABCG2 *gene, of which the most focused is an A to C substitution in exon 14 causing a change of the amino acid from tyrosin to serine [4,5]. This SNP (*ABCG2_49*) was first reported by Olsen et al. [4], who based on previous mapping data in Norwegian Red cattle excluded the SNP as being the most likely position of the QTL. In conflict to this result, Cohen-Zinder et al. suggested that the SNP (denoted *ABCG2 (2) *in reference [5]) is causative based on mapping results from the Holstein breed. A third strong candidate gene for the QTL is the *PKD2 *gene, which gene product is involved in calcium homeostasis. Since calcium is the major osmotic component in milk, differences in *PKD2 *expression could affect the content of water in the milk, and thus indirectly reduce milk fat and protein percentages. All three genes are expressed in the mammary gland.

Due to the conflicting results in different cattle populations, we repeated the analyses from [4] with a denser map including both *ABCG2_49 *and *OPN_3907*, as well as 25 SNPs in *PKD2*. Regions outside the *ABCG2 *to *LAP3 *region were also genotyped. As previously, a combined linkage and linkage disequilibrium analysis [3] was performed for protein and fat percentages.

The single-QTL analyses yielded rather similar results for the two traits, with the highest LRT values found for marker brackets in *ABCG2*, *PKD2 *and *OPN*. However, the test statistic had several peaks in a rather broad region, and the QTL position could not be further refined. In this case, where several different genes have been proposed as the causative gene, the many peaks may reflect the presence of more than one QTL. We utilized a multi QTL approach where each interesting marker was included in the QTL model in turn. By including *OPN_3907 *or any other marker except *ABCG2_49*, the logL ratio curve was similar to the curve of the single QTL analysis, indicating that these markers had minor effects on the trait. However, including *ABCG2_49 *in the model yielded a flat curve, indicating that this term explains most of the QTL variation. In fact, it seems to be the only genotyped marker with an effect, since if there were two QTLs, a peak in the region of the second QTL would remain when fitting the effect of *ABCG2_49*. Therefore, *OPN_3907 *and all other genotyped markers can probably be excluded as causative mutations. In our data, we observe strong LD between *OPN_3907 *and *ABCG2_49 *(r^2 ^= 0.34), which may explain the findings of Schnabel et al. [6].

*ABCG2_49 *yielded a much higher LRT when fitted as a single marker in the 'best LD marker test' compared to when its bracket was fitted as a QTL in the single QTL analysis. If *ABCG2_49 *is the causal mutation, this increase in LRT is expected since *ABCG2_49 *does not only explain which gametes are IBD but also which gametes are Alike-in-State, i.e. the fit of the model is improved. *OPN_3907 *showed the opposite; when a QTL was fitted in its bracket, it showed an even higher LRT than the bracket of *ABCG2_49*, which implies that the IBD probabilities for this bracket, that result from the markers surrounding this bracket, fits the QTL very well. But by also requiring *OPN_3907 *to be Alike-in-State, i.e. by fitting *OPN_3907 *as a single marker, the likelihood drops. This indicates that *OPN_3907 *and the causative mutation are not Alike-in-State. Also from this piece of evidence, *OPN_3907 *can be excluded as the causal mutation.

Concordance between the sires' marker genotypes and segregation status for the QTL were found for *ABCG2_49 *only. The probability of concordance by chance was found to be 0.0018 in our study and 0.00008 in the unrelated study of Cohen-Zinder et al. [4], and thus the product of the concordance probabilities is 1,44*10^-7^. Again, this result excludes *OPN_3907 *and strongly suggests *ABCG2_49 *as the causal mutation.

We have identified a 6 Mb long haplotype with negative effects on fat and protein percentages and positive effect on milk yield. This haplotype is 6 Mb long and includes approximately 60 of the genotyped markers. The deletion in *OPN_3907 *is found in this haplotype, but as it is also found in a second haplotype with no particular effect on the traits, *OPN_3907 *can be excluded as the causal mutation. The *C *allele of *ABCG2_49 *is found only in this extreme haplotype, and *ABCG2_49 *is the only genotyped marker in perfect disequilibrium with the QTL. However, a region of 6 Mb will also contain a large number of polymorphisms other than those included in our study, and theoretically, one of these could be the true causal mutation and *ABCG2_49 *merely a marker in perfect LD with it. Our results indicate that general LD in the region is quite low, with an average r^2 ^of 0.2 between markers situated 50 kb apart. Thus, perfect LD seems to be rare in our population, and this strengthens the hypothesis of *ABCG2_49 *as the causal mutation. Still, functional studies are needed to verify the causality.

As shown in Figure 5, *ABCG2_49 *is only in LD with *OPN_3907*, which is 116 kb away. *OPN_3907 *is also in LD with *PKD2_7141*, which is 67 kb away. One possible explanation of these markers showing high LD with each other but not with more closely linked markers, could be if the rare alleles of these markers were present on a haplotype that were imported into Norwegian Red cattle from an other breed. If this is the case, the rare alleles on this haplotype show high LD, whereas the importation has little effect on markers with intermediate allele frequencies whose LD thus remain low. For instance, the *C *allele of *ABCG2_49 *segregate at a low frequency in US Holstein and British Friesian [13], and semen from these breeds have been used in the breeding program for Norwegian Red.

In our previous paper, we erroneously excluded *ABCG2_49 *as the causal mutation because animals carrying an extreme haplotype seemed to carry both the *A *and *C *alleles of the marker. Although the animal material was larger in that study, the map density in the QTL region was much lower, and it is possible that some haplotypes were estimated incorrectly. Confidence intervals of QTL positions are difficult to obtain, and a wrong choice of intervals could lead to wrong conclusions. However, the concordance of all results in our present paper with the results of Cohen-Zinder et al. [5] strongly suggests *ABCG2_49 *as the underlying mutation.

To sum up, six pieces of evidence are suggesting *ABCG2_49 *as the causal mutation: (i) The QTL affecting protein percentage is completely removed by correcting the QTL analysis for the effect of *ABCG2_49*. (ii) Concordance between segregation status of the sires and marker genotype is found for *ABCG2_49 *only. (iii) We have identified a 6 Mb long haplotype with negative effects on fat and protein percentages and positive effect on milk yield. *ABCG2_49 *is the only marker in perfect disequilibrium with the QTL. (iv) *ABCG2_49 *is the most likely candidate in two different populations, i.e. Norwegian Red cattle and Holstein population investigated by Cohen-Zinder et al. [5]. For both populations, the A allele is associated with higher fat and protein percentages and lower milk yield than the C allele. The allele substitution effects were also very similar in the two populations, corresponding to approx. 0.15% protein and 0.14% fat in Norwegian Red cattle, and 0.13% protein and 0.09% fat in the Holstein population. (v) The [A/C] mutation in *ABCG2_49 *is a missense mutation causing a tyrosine to serine substitution (Y581S) in the fifth extracellular region of the protein, which might affect the transporter function of the gene [5]. (vi) *ABCG2 *is expressed in the mammary gland and showed significant differential expression during lactation as compared to the dry period [5]. Together, these pieces of evidence provide strong support for the conclusion that *ABCG2_49 *is in fact the causative mutation.

Thus, using our dense marker map, all genetic analyses point to *ABCG2_49 *as the causative mutation. Functional analysis will be necessary to confirm these genetical findings. In any case, the diverging results of Schnabel et al. and Cohen-Zinder et al. as well as the results in the present study illustrate the power of confirmation of QTL mapping results across breeds to zoom in on the causative mutation.

## Conclusion

The present study shows that OPN_3907 can be excluded as the polymorphism underlying the QTL for fat and protein percentage on bovine chromosome 6. The results of this and other papers strongly indicate that the [A/C] mutation in *ABCG2_49 *is the causal mutation. However, the possibility that *ABCG2_49 *is only a marker in perfect LD with the true mutation cannot be completely ruled out.

## Methods

### Data

All animals in the study belonged to the Norwegian Red cattle breed. The animals were organized in a granddaughter design consisting of 18 elite sire families. The total number of sons in the study was 716, ranging from 24 to 69 sons for the smallest and largest families, respectively. The total number of daughters was approximately 507,000, with an average of 708 daughters per son. These families were only partly identical to those used in our previous paper [4]. We are currently performing QTL studies on BTA6 for a large number of traits, and the current families were chosen based on sufficiently large family sizes and/or availability of trait data. The pedigree of each animal in the study was traced back as far as known. Predicted transmitting abilities (PTAs) of the sons from the 18 families were used as performance information in the analyses. The PTAs for protein precentage and fat percentage were available from the national genetic evaluation in June 2000 carried out by GENO Breeding and AI Association, and evaluated using BLUP with a single trait sire-maternal grandsire model.

### Marker Map

We developed a dense marker map consisting of 403 Single Nucleotide Polymorphisms (SNPs) covering the entire length of bovine chromosome 6 [14]. A subset of this map, consisting of 91 SNPs was utilized in the present study. A description of the SNPs, including accession numbers in dbSNP, assays for genotyping on the MassARRAY system (Sequenom, San Diego, USA), marker allele frequencies and predicted physical distances between markers are found in Additional file 1. This map spans a region of 16.7 Mb and covers both the previously reported QTL region and flanking regions. Physical distances between markers were determined by BLAST search against sequences of BAC clone RPCI42_5K14 [GenBank:AJ871176] and contigs available from the 6× whole genome shotgun (WGS) assembly of the bovine genome [15]. In cases where bovine information was unavailable, coordinates to the comparative region on human chromosome 4 were found using BLAT [16] and used to predict physical distances between SNPs. As very few recombinations were found between the closely linked markers, genetic distances based on recombination rates could not be obtained. Instead, distances in Morgans were approximated by setting 1 cM equal to 1 Mb. As the statistical analyses required some recombination between markers, all small marker distances were increased to 0.0001 M. Marker names and positions are shown in Additional file 1.

### Single QTL analysis

Protein percentage and fat percentage was first analyzed separately using the combined linkage and linkage disequilibrium method of MEUWISSEN et al. [3]. In short, the method consisted of the following steps: First, the linkage phases of all sires and sons were estimated based on marker information. Second, the IBD probabilities of pairs of haplotypes were calculated using marker and pedigree information (for details, see [3] and [17]) at the midpoint of each marker bracket, which was regarded as the putative position for a QTL. Only the bracket midpoints were considered, since for a dense marker map individual positions within the bracket would have similar probabilities. The IBD probability depends on the effective population size and the number of generations since the base population. However, since the QTL position estimates are found to be rather insensitive to the choice of effective population size and number of generations [18], these were both assumed to be 100. The haplotype window used for computation of IBD probabilities included all 91 markers. The matrix of IBD probabilities between the haplotypes at position i is denoted **G**_**i**_. The last step was to compare the correlations in the **G**_**i **_matrix to that in the data using a residual maximum likelihood (REML) analysis. The statistical model used for this analysis was

**y **= μ**1 **+ **Zh **+ **u **+ **e**;

where **y **is a (n × 1) vector of records (i.e. PTAs for the trait in question), μ is the overall mean, **1 **is a vector of 1's, **h **is a vector of random haplotype effects of dimension q × 1, where q is the number of different haplotypes, **Z **is a (n × q) incidence matrix relating observations and haplotype effects, **u **is a vector of random polygenic effects and **e **is a vector of residuals. The variances of **h**, **u **and **e **are **G**_**i**_σ^2 ^_h_, **A**σ^2^u and **R**σ^2 ^_e_, respectively, where **G**_**i **_is the matrix of IBD probabilities among haplotypes, **A **is the additive genetic relationship matrix and **R **is a diagonal matrix with nj^-1 ^on the diagonals (nj is the number of daughters of bull j). For each marker bracket, the log-likelihood of a model containing the QTL (LogL(**G**_**i**_)) was calculated as well as a model fitting only background genes (LogL(0)) using the ASREML package [19]. A Likelihood Ratio Test-statistic (LRT) was calculated as LRT = LogL(**G**_**i**_) - LogL(0). The marker bracket with the highest LRT was expected to contain the QTL.

### Multi QTL analysis

A complete multi QTL analysis could not be performed due to convergence problems caused by the highly correlated **G**_**i **_matrices, which were due to the small bracket sizes. Instead, we utilized the same analysis as for the single QTL analyses, but included also a random effect of a specific marker. Markers regarded as putative QTL from the single QTL analyses (e.g., markers situated in the critical region and with a high LRT) were included in the model in turn, and the QTL analysis was repeated. These analyses search for additional QTL, given that the QTL at the marker is accounted for, and is similar to the fitting of cofactors [20].

### Best LD marker

The LRT of each separate marker was found by analysing the data using a model containing a random effect of the marker alleles as well as the mean and a random animal effect, i.e. the marker was fitted as **h **in model [1] assuming its allelic effects were independent (**G**_**i **_= **I**). The marker with the highest LRT is in highest LD with the QTL (or alternatively the causative mutation itself), and is subsequently 'denoted the best LD marker'.

### Concordance between marker and QTL genotypes

Sires segregating for the QTL were found from the single QTL analysis, which provided estimates of the effect of the QTL alleles in each putative QTL position. Probability of concordance by chance between the QTL and a polymorphism was computed only for *ABCG2_49 *using the formula p^nhet^*(1-p)^nhom^, where p is the probability of heterozygotes (which is found from the allele frequencies of *ABCG2_49*), nhet is the number of sires segregating for the QTL and nhom is the number of sires not segregating for the QTL.

### Haplotype analysis

Next, we tried to find haplotypes that could explain the QTL effect. Starting at a marker position that showed a high association with the QTL (i.e. fitting the marker alone yielded a high likelihood), the haplotypes were extended to the left or to the right with one marker at a time. The haplotype was extended to the left if this gave a higher likelihood than extending it to the right and vice versa. The haplotype search stopped if the extension to the left and right did not yield a higher likelihood than that of the current haplotype. Model [1] was also used for this analysis, where **h **is now the effect of the above haplotype, and haplotype effects were assumed independent, i.e. **G**_**i **_= **I **(the identity matrix). Both paternal and maternal haplotypes were included in the analysis. The paternal haplotypes come from few sires and could theoretically affect the haplotype frequencies. However, this is also what is found in the real population, i.e. few sires will increase the frequency of particular haplotypes considerably. For the estimation of haplotype effects, both the paternal and maternal haplotypes are accounted for since the records are affected by both. The paternal haplotypes might occur very often together with the background genes of a particular sire, but this was corrected for by including a polygenic effect in the model, which takes account of the effect of the background genes.

### LD and haplotype block structure of the QTL region

Haplotypes detected by the above-mentioned method were imported into the Haploview program [10] for calulation of LD (r^2^) between markers and construction of haplotype blocks using the algorithm of Gabriel et al. [11].

## Authors' contributions

HGO performed the statistical analyses and drafted the paper.

HN was performing the SNP detection and was involved in the map construction.

BH designed the script for multidesign of PCR primers, established the SNP detection programs and helped to draft the paper.

PRB supervised the MassARRAY genotyping.

MS provided all pedigree and performance information.

SL was coordinating the SNP identification and genotyping process, constructed the map and helped to draft the paper.

TM developed all statistical procedures and helped to draft the paper.

All authors read and approved the final manuscript.

## Supplementary Material

Additional file 1 **Marker information**. Accession numbers, marker names, positions, allele frequencies in the genotyped animals, LRTs for protein and fat percentages from the 'best LD marker test', primer sequences and a 100 bp string containing the SNP. Click here for file
